# Exploration of the Strategy for Improving the Expression of Heterologous Sweet Protein Monellin in *Aspergillus niger*

**DOI:** 10.3390/jof9050528

**Published:** 2023-04-29

**Authors:** Ke Li, Junwei Zheng, Leyi Yu, Bin Wang, Li Pan

**Affiliations:** School of Biology and Biological Engineering, South China University of Technology, Guangzhou Higher Education Mega Center, Guangzhou 510006, China; 202020148715@mail.scut.edu.cn (K.L.); 20710106321@mail.scut.edu.cn (J.Z.); 201911008179@mail.scut.edu.cn (L.Y.); btbinwang@scut.edu.cn (B.W.)

**Keywords:** *Aspergillus niger*, monellin, heterologous expression, HiBiT-tag

## Abstract

*Aspergillus niger* is a primary cell factory for food-grade protein (enzyme) production due to its strong protein secretion capacity and unique safety characteristics. The bottleneck issue for the current *A. niger* expression system is the difference in expression yield of heterologous proteins of non-fungal origin compared to those of fungal origin, which is about three orders of magnitude. The sweet protein monellin, derived from West African plants, has the potential to become a food-grade sweetener due to its high sweetness and the benefit of not containing sugar itself, but it is extremely difficult to establish a research model for heterologous expression in *A. niger*, owing to extremely low expression, a small molecular weight, and being undetectable with conventional protein electrophoresis. HiBiT-Tag was fused with low-expressing monellin in this work to create a research model for heterologous protein expression in *A. niger* at ultra-low levels. We increased monellin expression by increasing the monellin copy number, fusing monellin with the endogenous highly expressed glycosylase *glaA*, and eliminating extracellular protease degradation, among other strategies. In addition, we investigated the effects of overexpression of molecular chaperones, inhibiting the ERAD pathway, and enhancing the synthesis of phosphatidylinositol, phosphatidylcholine, and diglycerides in the biomembrane system. Using medium optimization, we finally obtained 0.284 mg/L of monellin in the supernatant of the shake flask. This is the first time recombinant monellin has been expressed in *A. niger*, with the goal of investigating ways to improve the secretory expression of heterologous proteins at ultra-low levels, which can serve as a model for the expression of other heterologous proteins in *A. niger*.

## 1. Introduction

*Aspergillus niger* is a food spoilage fungus that can be found naturally in various agricultural products across a wide range of temperatures (6–47 °C) and pH levels (1.5–9.8) [1]. This fungus has a strong protein secretion capacity in which a large number of industrial enzymes are produced, such as glucoamylase, amylase, glucose oxidase, pectinase, peroxidase, phytase, xylanase, and cellulase, among others [2]. Moreover, *A. niger* has unique safety properties, and many of its products are classified as GRAS (Generally Recognized as Safe) by the U.S. Food and Drug Administration. It can also thrive and multiply in low-cost biomass materials, resulting in significant industrial production cost reduction [3,4,5]. These remarkable properties make *A. niger* one of the best hosts for the expression of edible enzymes and food-grade proteins.

Although *A. niger* has the capability of producing diverse and copious amounts of proteins and enzymes, the expression of heterologous proteins that are not of fungal origin is only possible at the “mg/L” level, while homologous proteins can be expressed at the “g/L” level. For instance, while *A. niger* can produce 25–30 g/L of glucoamylase in a shake flask culture [6], the expression yield for heterologous protein was only 70 mg/L [2,4]. The final yield of a heterologous protein is influenced by many factors, such as transcription, translation, post-translational modifications, protein transport, protein hydrolases, and certain degradation compounds in the culture medium. Heterologous proteins need to undergo numerous stages before reaching the extracellular medium, such as transportation to the endoplasmic reticulum (ER) for folding and maturation, processing at the Golgi apparatus, and transportation to the cell membrane by fusion with vesicles. Finally, heterologous proteins need to cross the cell wall to reach the cytoplasmic medium [7]. The efficiency of each stage can significantly influence the final extracellular protein yield, and each stage can be influenced by various factors. The main influencing factors at the transcriptional stage include promoter strength, activation of transcription factors, the copy number of protein genes, and the position of protein genes in the genome. During the translation and processing stage, the major influences include host codon preference, signal peptide recognition, the unfolded protein response (UPR), the endoplasmic reticulum-associated protein degradation pathway (ERAD), and autophagy (AP). Finally, secretion into the extracellular compartment, the main influence during this phase, is vesicular transport between the Golgi apparatus and the cytoplasmic membrane [8].

Numerous approaches have been suggested to enhance the yield of proteins, such as (1) utilizing strong endogenous promoters and signal peptides to boost protein expression [9,10,11]; (2) upregulating the transcriptional activator *amyR* to markedly improve glycosylase and amylase activity by 15-fold [12]; (3) increasing the target gene’s copy number [13,14]; (4) fusing the target protein to a host with a high expression of endogenous proteins [15,16]; (5) enhancing the unfolded protein stress response (UPR) of the host [17,18,19]; (6) reducing the endoplasmic reticulum-associated protein degradation pathway (ERAD) [20,21]; (7) circumventing the degradation of heterologous proteins by endogenous proteases [22,23]; and (8) manipulating the morphology and optimizing the fermentation process of *A. niger*, among others [2,7]. Nonetheless, it is still challenging to achieve high yields of heterologous proteins in *A. niger*, particularly for small heterologous proteins that are less closely related.

In recent years, as obesity, diabetes, and hyperlipidemia rates surge due to high sugar consumption, the search for novel, low-calorie sweeteners has become a prominent area of interest. Monellin is a small, sweet heterodimeric protein originally isolated from the West African plant *Dioscoreophyllum cumminsii*, which is 3000 times sweeter than sucrose for the identical mass [24,25,26]. Composed of a 44-amino acid residue A chain and a 50-residue B chain, this compact globular protein (~11 kDa) loses its sweetness at about 50 °C and lower pH levels [25]. To improve its thermal stability while retaining the same level of activity and sweetness as the native protein at extreme pH and high temperatures, researchers developed a single-chain monellin (MNEI), which links the two natural chains via a Gly-Phe dipeptide junction [27]. Subsequently, expression and targeted mutagenesis of monellin mostly relied on MNEI. However, the natural monellin sweet protein extracted from plants cannot meet the demand of the food industry, and further research into a heterologous expression is required. Unfortunately, the small molecular weight and ultra-low expression of monellin make it difficult to be detected using conventional protein detection methods. HiBiT is a 1.3 kDa peptide (11 amino acids) that generates bright quantitative luminescence by high-affinity complementation with an 18 kDa subunit derived from NanoLuc (LgBiT). The HiBiT-tag can be attached to any protein, facilitating highly sensitive and quantitative bioluminescence detection without the need for antibodies [28].

In this study, a bioluminescence-based HiBiT-Tag was fused with codon-optimized monellin to create a model for investigating the expression of heterologous proteins at ultra-low levels in *A. niger*. The expression of monellin was increased in terms of increasing its copy number, fusing monellin with the endogenous, highly expressed glycosylase *glaA*, and eliminating extracellular protease degradation. Furthermore, this study explored the effects of overexpressing molecular chaperones, attenuating the ERAD pathway, and enhancing cellular biofilm phospholipid class synthesis on monellin expression. Using medium optimization, monellin was obtained in the supernatant of a shake flask at 0.284 mg/L. In this study, recombinant monellin was expressed in *A. niger* for the first time, with the aim of exploring methods to enhance the secretory expression of heterologous proteins, which can provide a reference for the expression of other heterologous proteins in *A. niger*.

## 2. Materials and Methods

### 2.1. Strains and Media

The components and contents of the medium used in this study are shown in Table S1 [29]. All materials were autoclaved at 115 °C for 20 min before use. The strains of *A. niger* used in this study are listed in Table 1. All strains were grown at 30 °C, incubated in CD medium at rest, and grown in DPY medium or starch fermentation medium at 220 rpm.

### 2.2. Construction of Recombinant Plasmids

The amino acid sequence and codon-optimized nucleotide base sequence of the single-stranded monellin expressed in this study are shown in Table S1. The nucleotide base sequence was synthesized by the company (GenScript, Nanjing, China), and the 8×His-tag and HiBiT-tag were added to the N-terminal and C-terminal ends of monellin, respectively. The signal peptide of *glaA* in the WT (SH-2∆*kusA*, ∆*pyrG*, GenBank assembly accession: GCA_000633045.1) strain was used for monellin expression to facilitate monellin secretion (Table S2). The expression cassette plasmids and CRISPR/Cas9 plasmids used in this study are listed in Table 2. The DNA fragments were amplified using the genomic DNA of strain WT as a template for the expression cassette vector construction, and the fragments were amplified with PrimeSTAR^®^ Max DNA Polymerase (Takara, Japan). The vectors were ligated using the Clone Smarter TOPO Cloning Kit according to the manufacturer’s instructions. The constructed vectors were cloned in *Escherichia coli* Mach1 T1, and the plasmids were extracted with the Plasmid Extraction Kit (Magen, Guangzhou, China) before the transformation.

### 2.3. Genetic Regulation of A. niger Based on the CRISPR/Cas9 System

The site-specific gRNAs used in this study were predicted and designed with CRISPR RGEN Tools (http://www.rgenome.net/cas-offinder/, accessed on 19 March 2022). PAfU6 was used as the promoter for sgRNA generation and TU6 as the terminator, forming PAfU6-sgRNA-TU6 (Figure S1). The primers for CRISPR/Cas9 plasmid construction are listed in Table S2. The pFC330 plasmid (carrying the spCas9 expression cassette and Af*pyrG* marker) treated with PacI restriction endonuclease was used as a ligation vector for CRISPR/Cas9 plasmid construction, and the pFC332-Dual locus plasmid [13] (carrying the spCas9 expression cassette, pFC332-PAOU6-glaAsgRNA-TU6-PAfU6-amyAsgRNA-TU6) was used as a template for fragment amplification. Before the transformation, *A. niger* strains were grown in DPY medium for 2 days to obtain viable mycelial cells. The transformation of *A. niger* was achieved using the PEG-mediated protoplast transformation method that has been described [32].

The CRISPR/Cas9 plasmids (pFC330 series plasmids) carried Cas9 and sgRNA, which were used to generate double-strand breaks (DSBs) at specific chromosomal loci, and then the cyclic plasmids carrying the repair fragments were integrated into the specific chromosomal loci using homologous recombination. The CRISPR/Cas9 plasmid and the cyclic plasmid were co-transformed into *A. niger* with 5 μg and 50 μg, respectively. After the successful acquisition of the transformants, the CRISPR/Cas9 plasmids can be easily eliminated by passing the transformant through three generations of culture under the selective pressure of 5-fluoroorotic acid (5-FOA) and uracil nucleoside (U).

### 2.4. RNA Extraction and Quantitative Real-Time PCR (qRT-PCR) Analysis

Exponential phase *A. niger* mycelia were collected and thoroughly ground under freezing in liquid nitrogen, and then total RNA was extracted using the HiPure Fungal RNA Kit (Magen, Guangzhou, China) and reverse transcribed using the PrimeScript RTPCR Kit (TaKaRa, Dalian, Japan) according to the manufacturer’s instructions. The gene-specific primers used for qRT-PCR analysis are listed in Table S4. Glyceraldehyde-3-phosphate dehydrogenase (*gpdA An16g01830*) and the actin structural protein (*actA An15g00560*) were used as the internal reference gene, the qRT-PCR reagent was 2×SG Fast qPCR Master Mix (High Rox, BBI), and the instrument used was the QuantStudioTM 1 Plus Fluorescence quantitative PCR instrument (ABI/Thermo Fisher, Waltham, Massachusetts, USA). The mixing reagents and cycling conditions for qRT-PCR are shown in Tables S5 and S6, respectively.

### 2.5. Characterization of Monellin Expression

We used the Nano-Glo^®^ HiBiT Extracellular Assay System (Promega, Madison, Wisconsin, USA) to measure the luminescence values of the shake flask supernatant. First, based on the number of samples, we calculated the amount of Nano-Glo^®^ HiBiT extracellular reagent required. For example, if we have 6 samples and 3 technical parallel experiments are performed for each sample, then a total of 18 luminescence values have to be detected. Taking into account some losses when transferring the liquid, we calculated the amount using 20 detected luminescence values, which requires 20 × 50 µL of Nano-Glo^®^ HiBiT extracellular reagent, i.e., 1000 µL. Then, we diluted the LgBiT protein and Nano-Glo^®^ HiBiT extracellular substrate at 1:100 and 1:50 into the appropriate amount of room temperature Nano-Glo^®^ HiBiT extracellular buffer in a new tube and mixed upside down. Finally, we aspirated 50 µL of shake flask supernatant and 50 µL of Nano-Glo^®^ HiBiT extracellular reagent into a 96-well light-proof enzyme labeling plate and left it for 5 min at room temperature before detecting the luminescence with Multiscan Spectrum (Tecan, Mannedorf, Switzerland). After that, we measured the amount of total secreted protein in the shake flask supernatant using the BCA protein quantification kit (GBCBIO, Guangzhou, China) according to the manufacturer’s instructions. In this study, the ratio of luminescence to total secreted protein amount was used to characterize monellin expression.

### 2.6. Quantification and Blotting of Monellin

The HiBiT Control Protein (Promega, Madison, WI, USA) is a 20 μM solution of purified recombinant 36 kDa HaloTag^®^ protein fused at its carboxy terminus to the 11-amino-acid HiBiT tag. The 20 μM HiBiT Control Protein was diluted 2000 times with the supernatant from the starch fermentation medium to obtain a solution of 10^−8^ M. Nine gradients were then diluted between 10^−8^ M and 10^−9^ M. The luminescence of each gradient was then detected to plot the calibration curve. A Tris-tricine-SDS-PAGE gel preparation kit (Solarbio, Beijing, China) was used for protein separation of the shake flask supernatant. After monellin was separated using Tris-tricine-SDS-PAGE, it was transferred to a PVDF membrane at 350 mA for 30 min. Protein blotting was performed using the Nano-Glo^®^ HiBiT Blotting System (Promega, Madison, WI, USA) according to the manufacturer’s instructions, and then the PVDF membrane was transferred to a gel multifunction imager and imaged after 5 min of exposure.

## 3. Results

### 3.1. Establishment of the Monellin Expression Model

**Figure 1:**
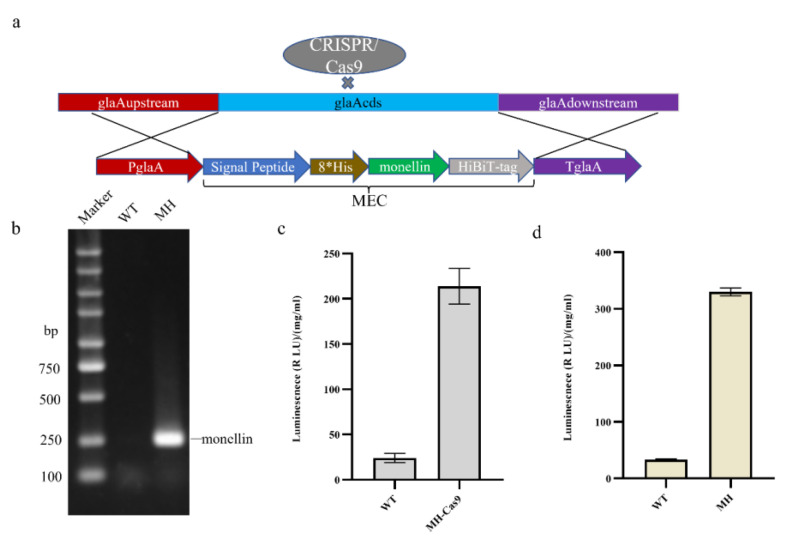
Construction of a heterologous expression model for monellin in *Aspergillus niger*. (**a**) Schematic representation showing monellin knocked into the *glaA* locus with homologous recombination. (**b**) Verification that monellin was successfully transcribed. (**c**) Expression of monellin in MH carrying the CRISPR/Cas9 plasmid. (**d**) Expression of monellin in MH after the CRISPR/Cas9 plasmid was eliminated. (Three biological replicates and three technical replicates were used).

To construct an expression model for monellin detection, we attached HiBiT-tag (11 amino acids) to the C-terminus of monellin, as shown in Figure 1a, and knocked the monellin expression cassette into the *glaA (An03g06550)* site using CRISPR/Cas9 Homologous Direct Repair (CRISPR-HDR) technology with P*glaA* as the promoter. Co-transformation of 50 µg monellin expression cassette from cyclic plasmid and 5 µg pFC330-1 plasmid was conducted, starting with a WT (*SH-2* ∆*kusA*, ∆*pyrG*) strain. Identification of target transformants was achieved with gene positioning upstream and downstream (Figure S2). RNA extraction and reverse transcription from the transformants yielded cDNA, with successful transcription of monellin confirmed with PCR amplification using monellin identification primers (Table S7), as demonstrated in Figure 1b. The transformants were cultured in a CD medium for 7 days, and then 5 mL were inoculated into starch fermentation medium for 6 days to measure monellin expression (Figure 1c). The CRISPR/Cas9 plasmid was eliminated with the selective pressure of 5-fluoroorotic acid (5-FOA) and uracil nucleoside (U), after which the monellin expression in the strain was further measured (Figure 1d). Consequently, we successfully detected monellin in the supernatant from the shake flask and obtained MH as a host strain for further studies. Interestingly, monellin expression seemed to be higher when the CRISPR/Cas9 plasmid was eliminated. We speculated that the presence of the nuclease Cas9 is a little toxic to the *A. niger* strain, affecting its growth and leading to a little higher expression of monellin.

### 3.2. Increasing the Copy Number of Monellin to Enhance Its Expression

**Figure 2:**
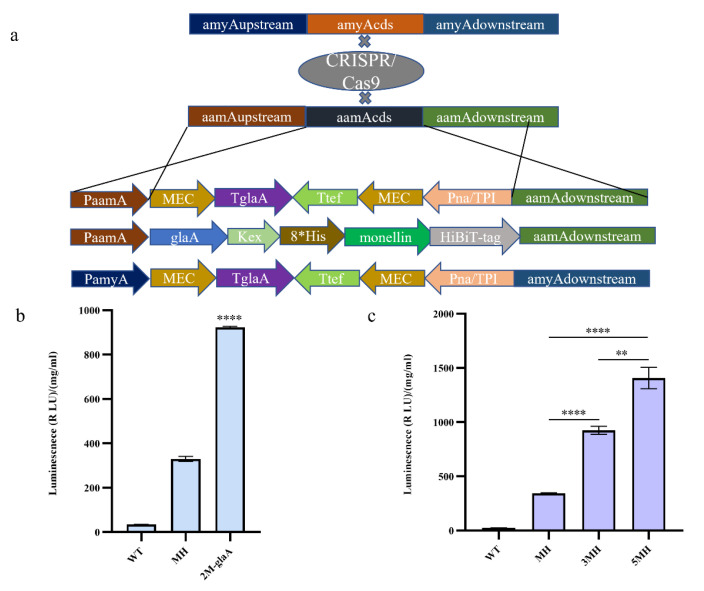
Increasing the copy number of monellin and its fusion with *glaA* to increase the expression of monellin. (**a**) Schematic diagram showing the monellin double copy-plasmid and vector for the fusion of monellin with *glaA*. (**b**) Expression of monellin in strains of monellin fused with *glaA*. (**c**) Expression of monellin in strains with different monellin copy numbers. (**** means *p* < 0.0001, ** means *p* < 0.01, and these indicate a significant difference compared with the control. Three biological replicates and three technical replicates were used).

To explore whether increasing the copy number of monellin could increase its expression, we constructed expression cassettes carrying double copies of monellin for knocking into the *aamA (An11g03340)* locus and *amyA (An05g02100)* locus, respectively (Figure 2a). We inserted the expression cassette containing two copies of monellin into the *aamA* locus of the MH strain, upon which we obtained the 3MH strain following the removal of the CRISPR/Cas9 plasmid. For monellin expression, we obtained a 2.70-fold increase in 3MH compared to MH (Figure 2c). Additionally, we fused monellin to the active part of the *glaA* in *A. niger* (the first 514 amino acid residues of *glaA*) into an expression cassette (Figure 2a), and the glycosylase was linked to monellin with a Kex-linker (AATGTGATTTCCAAGCGCAAGCGC). We knocked the expression cassette into the *aamA* locus and obtained strain 2M-glaA. The results indicated that the fusion of monellin with *glaA* yielded a 2.8-fold increase in monellin expression when compared to the MH strain (Figure 2b). To further understand the effect of increasing the copy number of monellin on its expression, we then knocked the expression cassette into the *amyA* locus and obtained 5MH after eliminating the CRISPR/Cas9 plasmid. The expression of monellin was augmented by 1.52-fold in 5MH compared to 3MH (Figure 2c). We concluded that increasing the copy number did increase monellin expression, and the increase in expression became smaller as the copy number of monellin increased.

### 3.3. Effects of Overexpression of Key Genes in the Phospholipid Synthesis Pathway and the UPR Pathway on Monellin Expression

In fungi, the regulation of phospholipid metabolism is a specific regulatory process that mainly includes fatty acid biosynthesis, phospholipid biosynthesis, and accumulation. These processes also affect protein transport, proteolytic metabolic processes, and proteolytic reactions resulting from amino acid starvation [33]. The basic helix-loop-helix (bHLH)-type phospholipid regulatory transcription factor *ino2 (An02g04350)* was identified in *A. niger*, and defects of *ino2* were found to cause an imbalance in total cellular phosphatidylcholine [34]. Furthermore, *Opi3 (An08g00560)* plays a pivotal role in the synthesis of phosphatidylcholine (PC) from phosphatidylethanolamine (PE) [35], while phosphatidic acid phosphatase *pah1 (An01g12610)* catalyzes phosphatidic acid (PA) into diglycerides (DAG), a precursor for PE, PC, and phosphatidylinositol (PI) synthesis [36], which is important for maintaining total cellular phospholipid levels. In addition, overexpression of the molecular chaperone *sed5* promoted the intracellular vesicle transport process in yeast, thereby increasing the expression of secreted proteins [37].

**Figure 3:**
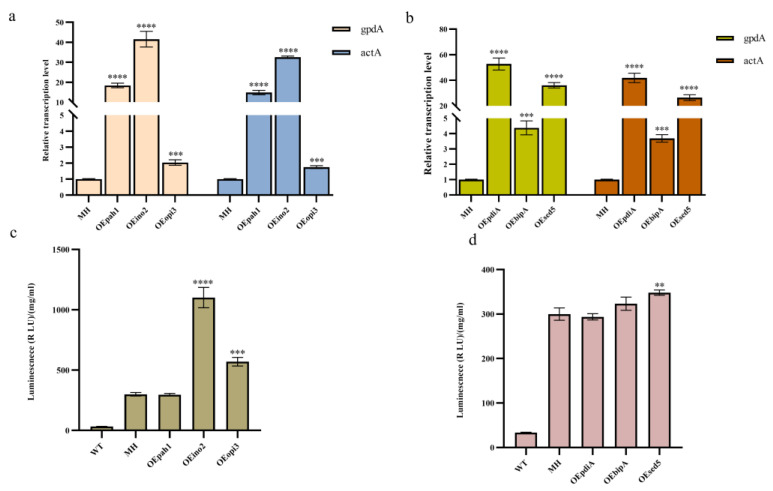
Effects of gene overexpression in MH on monellin expression. (**a**,**b**) The relative quantification of qRT-PCR for the strains overexpressing phospholipid synthesis-related genes and molecular chaperone genes, respectively. (**c**,**d**) The expression of monellin in strains overexpressing phospholipid synthesis-related genes and molecular chaperone genes on monellin expression, respectively. (**** means *p* < 0.0001, *** means *p* < 0.001, ** means *p* < 0.01, and these indicate a significant difference compared with the control. Three biological replicates and three technical replicates were used).

To explore the effects of overexpression of key genes in the phospholipid synthesis pathway and the UPR pathway on monellin expression, we overexpressed the key genes for phospholipid synthesis, *ion2*, *pha1*, *opi3*, the molecular chaperone *bipA (An11g04180)*, and the disulfide isomerase *pdiA (An02g14800)* in MH (Figure S2). Meanwhile, we found the homologous protein *sed5 (An02g12980)* of the molecular chaperone *sed5* in *A. niger* and overexpressed it in the MH strain as well. The qRT-PCR results (Figure 3a,b) indicated that all these genes were successfully overexpressed. We then examined monellin expression and concluded that overexpression of *ino2*, *opi3,* and *sed5* resulted in a 3.67-fold, 1.9-fold, and 16% increase in monellin expression, respectively (Figure 3c). In addition, there was no significant change in monellin expression when overexpressing *pha1*, *pdiA*, or *bipA* (Figure 3d).

### 3.4. Effects of Knocking out Key Genes in the Extracellular Protease and ERAD Pathways on Monellin Expression

**Figure 4:**
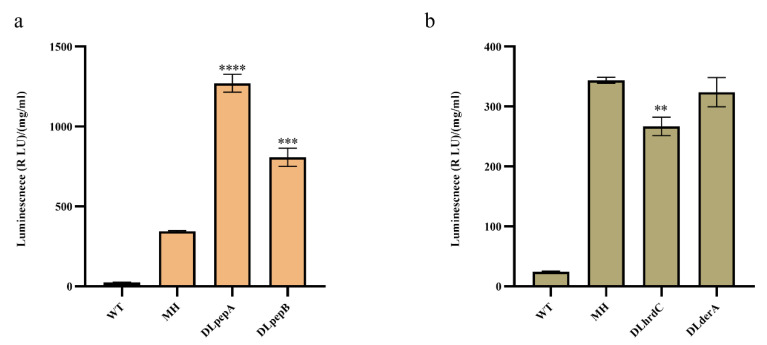
Effects of gene deletion in MH on monellin expression. (**a**,**b**) The effects of knocking out extracellular protease and key genes in the ERAD pathway on monellin expression, respectively. (**** means *p* < 0.0001, *** means *p* < 0.001, ** means *p* < 0.01, and these indicate a significant difference compared with the control. Three biological replicates and three technical replicates were used).

To deal with the degradation of monellin in the expression steps, we devoted our attention to extracellular proteases and the ER-associated protein degradation (ERAD) pathway in *A. niger*. In MH, we deleted the extracellular proteases *pepA (An14g04710)* and *pepB (An01g00530)* using CRISPR/Cas9-HDR technology (Figure S2), and in the same starting strain, we also attempted to delete the key genes *derA (An15g00640)* and *hrdC (An01g12720)* in the ERAD pathway. As illustrated in Figure 4, the deletion of *pepA* and *pepB* increased monellin expression by 3.7-fold and 2.35-fold, respectively. Deleting *derA* had no significant impact on monellin expression, while the knockout of *hrdC* surprisingly resulted in a 22.3% decrease in monellin expression. That is, monellin was subjected to substantial degradation by extracellular proteases after being secreted into the extracellular medium.

### 3.5. Effects of a Combination of Knockout Protease Genes and Overexpression of Key Genes for Phospholipid Synthesis on Monellin Expression

Based on previous experiments, we confirmed that deleting extracellular proteases *pepA* and *pepB*, overexpressing the key phospholipid synthesis gene *opi3*, increasing the copy number of monellin, fusing with *glaA*, and overexpressing transcription factor *ino2* all led to a significant increase in monellin expression. In this study, we combined three strategies of knockout, overexpression, and copy number increase in an attempt to achieve even higher levels of monellin expression. Additionally, we constructed CRISPR/Cas9 plasmid pFC330-8 (Table 2) carrying both *pepA*sgRNA and *pepB*sgRNA for simultaneous generation of double-strand breaks at both the *pepA* and *pepB* loci.

We attempted several different combinations in 5MH, including constructing single knockout strains DL*pepA*-2 and DL*pepB*-2 for *pepA* and *pepB*, respectively, and a double knockout strain DLpA-pB. We inserted the *ino2* overexpression cassette into the *pepA* locus to achieve *ino2* overexpression along with *pepA* deletion to obtain the DLpAOEin strain. In the same way, we knocked the *opi3* overexpression cassette into the *pepB* locus to obtain the DLpBOEop strain, which achieved overexpression of *opi3* and deletion of *pepB* at the same time. We also deleted *pepA* while the *opi3* overexpression cassette was knocked into the *pepB* locus, creating strain DLpA-pBOEop, which achieved double knockout of *pepA* and *pepB* while also overexpressing *opi3*. Parallelly, we obtained the strain DLpA-pBOEin by knocking the *ino2* overexpression cassette into the *pepA* locus while deleting *pepB*, resulting in the double knockout of *pepA* and *pepB* and the overexpression of *ino2*. Finally, we introduced the *ino2* and *opi3* overexpression cassettes into the *pepA* and *pepB* loci, respectively, to generate the DLpA-pBOEin-op strain. The relative transcription levels of *ino2* and *opi3* in each strain are shown in Figure S3.

**Figure 5:**
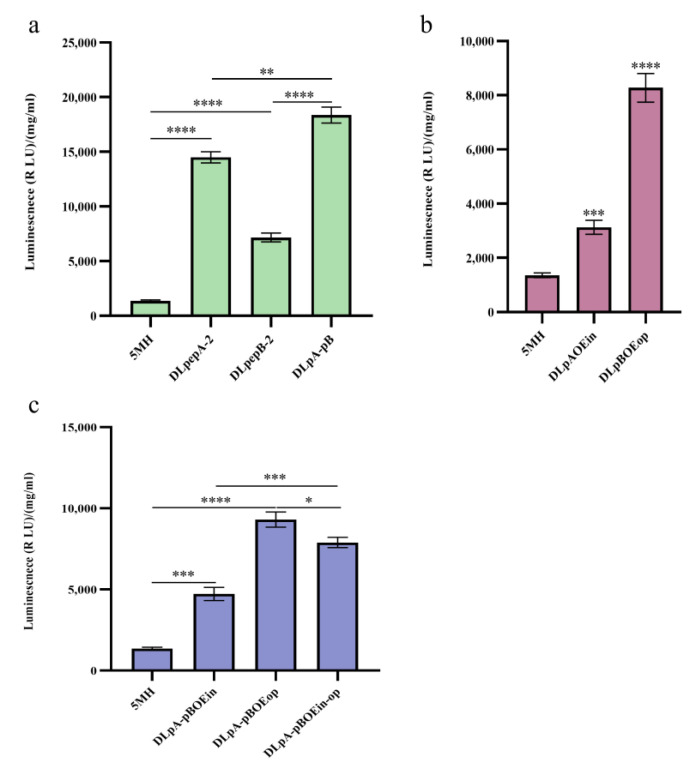
Effects of the combination of endogenous gene knockout and overexpression in 5MH on monellin expression. (**a**) The effects of knocking out the protease gene in 5MH on monellin expression. (**b**,**c**) The effects of the combination of knock-out protease and gene overexpression on monellin expression in 5MH. (**** means *p* < 0.0001, *** means *p* < 0.001, ** means *p* < 0.01, * means *p* < 0.05, and these indicate a significant difference compared with the control. Three biological replicates and three technical replicates were used).

The results (Figure 5a,b) showed that the expression of monellin in DL*pepA*-2, DL*pepB*-2, and DLpA-pB was 10.70-fold, 5.28-fold, and 13.54-fold higher, respectively, compared with 5MH. The expression of monellin in DLpBOEop was increased 6.10-fold; paradoxically, DLpAOEin was increased only 2.3-fold, even lower than DLpepA-2. Figure 5c indicated a 3.48-fold, 6.87-fold, and 5.8-fold increase in the expression of monellin in DLpA-pBOEin, DLpA-pBOEop, and DLpA-pBOEin-op, respectively, compared to 5MH. It was evident that deleting extracellular proteases had the greatest increase in improving monellin expression. Surprisingly, overexpressing *ino2* in the protease-deleted strain resulted in decreased monellin expression, and overexpressing *opi3* did not result in a considerable increase in secretory monellin expression.

### 3.6. Optimization of Fermentation Medium and Monellin–HiBiT-Tag Blotting

**Figure 6:**
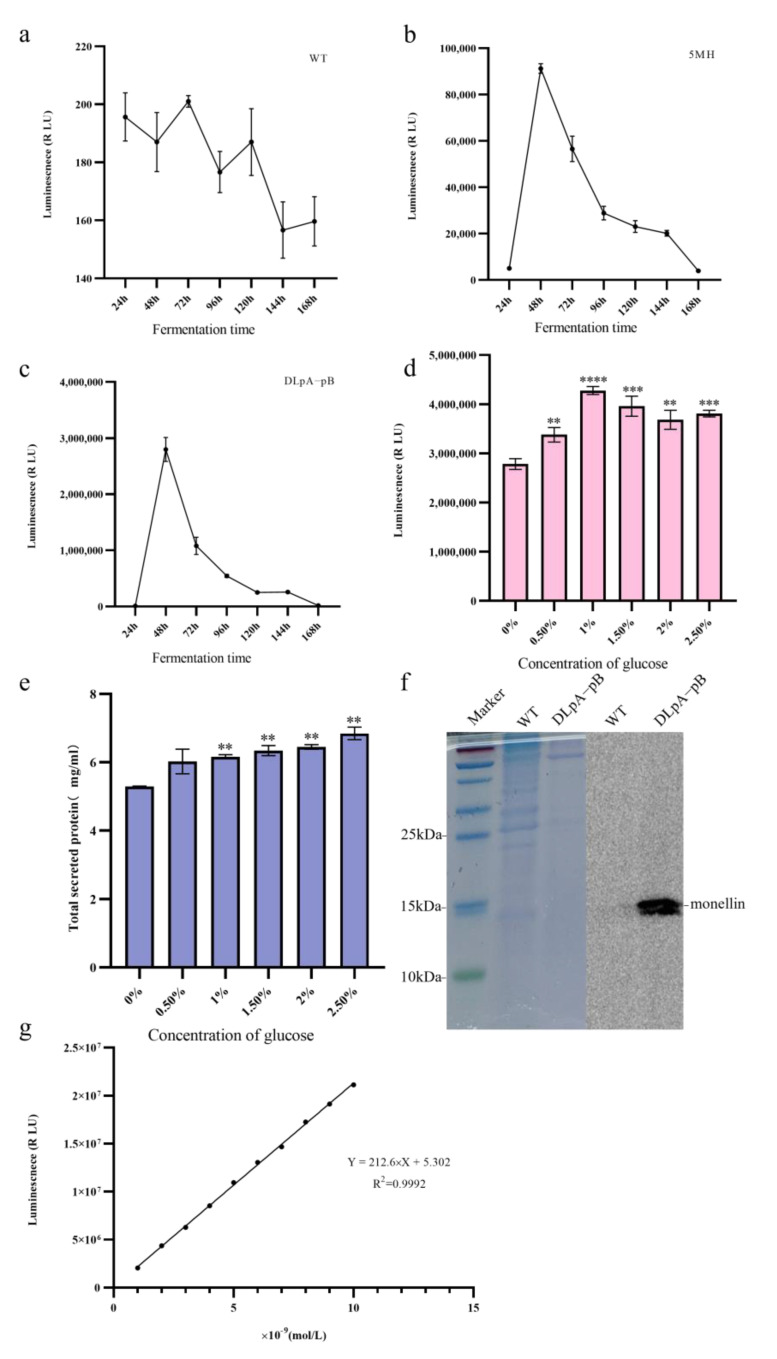
Effects of different fermentation times and glucose content on monellin expression. (**a**–**c**) The changes in monellin expression in WT, 5MH, and DLpA-pB with fermentation time, respectively. (**d**) The expression of monellin in DLpA-pB at different glucose concentrations. (**e**) The total amount of DLpA-pB secreted at different glucose concentrations. (**f**) Tris-Tricine-SDS-PAGE and HiBiT blotting experiments for the WT and DLpA-pB strains after 48 h of growth in an optimized fermentation medium. (**g**) Calibration curve for HiBiT-tag control protein (**** means *p* < 0.0001, *** means *p* < 0.001, ** means *p* < 0.01, and these indicate a significant difference compared with the control. Three biological replicates and three technical replicates were used).

We have established that the double knockout strain DLpA-pB, which lacks *pepA* and *pepB*, is the optimal strain for monellin expression. To investigate the variation in monellin secretion in *A. niger* during fermentation, we incubated the three strains (WT, 5MH, and DLpA-pB) in a starch fermentation medium and measured the luminescence in the supernatant every 24 h. Our results (Figure 6a–c) showed that monellin expression peaked at 48 h of incubation. 

As amylase deletion in 5MH may lead to reduced starch utilization in the fermentation medium, we compensated for this growth defect using DLpA-pB as the starting strain and added different concentrations of glucose to the original fermentation medium. After 48 h of fermentation, we detected the luminescence and total secreted protein of the fermentation supernatant. It can be seen that the luminescence and total secreted protein of the supernatant from the shake flask improved after the addition of glucose (Figure 6d,e). The addition of 1% glucose increased the luminescence of the shake flask supernatant by 53%. After Tris-Tricine-SDS-PAGE, we performed protein blotting experiments for monellin-HiBiT-Tag. Although monellin failed to be isolated with Tris-Tricine-SDS-PAGE, we successfully detected monellin in the monellin–HiBiT-Tag blot due to the high sensitivity of HiBiT-tag (Figure 6f). To accurately measure monellin in the supernatant from the shake flask, we established a calibration curve (Figure 6g) using diluted HiBiT Control Protein (see Section 2.6) and determined that the amount of monellin in the supernatant from the shake flask for DLpA-pB was approximately 0.284 mg/L. Table 3 revealed the expression of monellin in the different *A. niger* strains in this study compared to MH.

## 4. Discussion

*Aspergillus niger* produces a large number of industrial enzymes and is one of the most commonly used fungal cell factories. However, heterologous protein expression yields with it as a host are significantly lower than endogenous proteins, particularly for small heterologous proteins with distant relatives. Despite this, valuable enzymes from plant or animal sources still require industrial production, necessitating continuous exploration of strategies to improve heterologous protein expression in *A. niger*. Monellin, a plant-derived sweet protein, is only about 12 kDa and displays exceptional sweetness and a lack of sugar, making it a viable alternative to sweeteners. This study utilizes single-chain monellin as a heterologous expression indicator in *A. niger*, investigating various methods for enhancing heterologous expression.

First, for monellin, a protein with extremely low expression and undetectable enzymatic activity, we constructed a model for ultra-low expression in *A. niger* by fusing the HiBiT-tag to the C-terminus of monellin, taking advantage of the high affinity of two peptides of NanoLuc luciferase, i.e., LgBiT and HiBiT [28]. We also optimized the codon choice of monellin to improve protein expression secretion in *A. niger*, given that each organism tends to have a preferred codon use that influences multiple stages of protein biogenesis, including transcription, translation, co-translational folding, secretion, and post-translational modification [38]. Meanwhile, we selected the strong promoter P*glaA* in *A. niger* as the promoter for monellin expression and used its signal peptide to guide monellin secretion. Our efforts resulted in the successful detection of monellin in the shake flask supernatant from our basal strain MH (Figure 1d), after which various strategies were explored to boost monellin expression in this strain.

During the growth of *A. niger*, the production of proteolytic enzymes poses significant challenges to the expression of heterologous proteins. Silencing or deleting these protease genes helps to enhance the expression of heterologous proteins. For example, the knockdown of protease genes (*tppA*, *pepE*, *nptB*, *dppIV*, and *dppV*) in *Aspergillus oryzae* can effectively increase the expression of heterologous proteins, and the more protease genes are knocked down, the more effective is the effect [39]. The knockdown of *PrtT*, a transcription factor that regulates multiple proteases in *A. niger*, could promote the expression of recombinant proteins [40]. However, the knockdown of transcription factor *PrtT* can also have negative effects on the strain. The selection of culture conditions to reduce protease activity is not straightforward, as some of the factors involved are interrelated and may have unexpected antagonistic or synergistic effects [41]. The growth of the strain was severely affected when albumin and collagen were used as the only nitrogen sources [41,42]. In *Aspergillus fumigatus*, the knockdown of *XprG* and *PrtT* also affects the integrity of the cell wall [43]. Furthermore, even after knocking down *PrtT*, the strain still produced protease, which still had some degradation effect on the heterologous protein, suggesting that other transcription factors are involved in the expression of *A. niger*-secreted protease [40,41].

The protease *pepA* is one of the most important proteases in Aspergillus niger, accounting for 80–85% of its total protease activity [44]. Deletion of *pepA* in *Aspergillus awamori* increased the expression of Thaumatin [23]. Therefore, we generated protease knockout strains DL*pepA* and DL*pepB* using MH as the host, resulting in a 3.70-fold and 2.35-fold increase in monellin expression in DL*pepA* and DL*pepB*, respectively, compared to MH (Figure 4a). Building on this success, we then constructed protease-knockout strains DL*pepA*-2, DL*pepB*-2, and DLpA-pB using monellin multicopy strain 5MH as the host. Compared to strain 5MH, the expression of monellin in DL*pepA*-2, DL*pepB*-2, and DLpA-pB showed significant improvements, with a 10.70-fold, 5.28-fold, and 13.54-fold increase, respectively (Figure 5a). Our results demonstrated that knocking down the extracellular proteases *pepA* and *pepB* in *A. niger* led to a substantial increase in the expression of monellin, both in single-copy recombinant strains and multicopy recombinant strains. Moreover, we found that deleting *pepA* had a more substantial effect than deleting *pepB*, and the knockdown of both extracellular proteases led to increased monellin secretion. These findings indicate that the expression of the heterologous protein monellin faces significant challenges from extracellular proteases during its expression in *A. niger*.

In filamentous fungi, the ER can perform the initial processing of polypeptide chains from the ribosome, such as folding and assembly, but there is a limit to this processing capacity. When excessive amounts of polypeptides are transported to the ER, they can lead to their misfolding or delayed folding and assembly, leading to an unfolded protein stress response (UPR) [45]. The ER-associated protein degradation (ERAD) pathway in filamentous fungi is responsible for degrading misfolded or mispackaged proteins to ensure the correct structure of the final synthesized protein. However, when the UPR is triggered and is not able to process the unfolded proteins that remain in the ER promptly, the cell degrades these unprocessed proteins via ERAD, thereby relieving protein stress in the ER [7]. Based on the classical protein secretion pathway in filamentous fungi, we recognize that the ER is the critical site for the folding and processing of heterologous proteins. Therefore, we investigated whether the secreted expression of monellin was impacted by its folding and degradation in the ER. To enhance ER protein folding, we overexpressed *bipA* and *pdiA*, key genes in the UPR pathway, while deleting *derA* and *hrdC*, key genes in the ERAD pathway, to reduce the degradation of heterologous proteins by the ER, in an attempt to boost monellin expression. However, our results in Figure 3d and Figure 4b showed that these strategies did not lead to an increase in the secreted expression of monellin. Interestingly, the knockdown of *hrdC* reduced the monellin expression. As reported in a previous study [20], both the induction of the UPR and activation of the ERAD pathway require a significant amount of protein translocation into the ER. However, due to the extremely low expression of monellin, both pathways were not activated.

Lipids are essential components of the biofilm system and play a critical role in maintaining a dynamic balance within biofilms. As demonstrated in Figure 3c, overexpressing the key genes *pha1*, *ino2*, and *opi3* involved in phospholipid synthesis in *A. niger* (MH strain) resulted in a 0%, 3.67-fold, and 1.90-fold increase in the expression of monellin, respectively. To further investigate this result, we combined gene overexpression with protease gene deletion to construct DLpAOEin, DLpBOEop, DLpA-pBOEin, DLpA-pBOEop, and DLpA-pBOEin-op strains. Surprisingly, monellin secretion was significantly reduced by 3.63-fold in DLpAOEin compared to DLpepA-2, while it increased by 15.6% in DLpBOEop compared to DLpepB-2. The secretion of monellin in DLpA-pBOEin, DLpA-pBOEop, and DLpA-pBOEin-op was reduced by 2.89-fold, 0.97-fold, and 1.33-fold, respectively, based on DLpA-pB (Figure 5). Our findings suggest that in the *pepA* knockout strain, overexpressing *ino2* or *opi3* had a negative impact on the secretory expression of monellin. However, overexpressing *opi3* in the *pepB* knockout strain did not significantly affect the expression of monellin. The potential mutual influence mechanism between *pepA* and phospholipid metabolic pathways in *A. niger* remains unknown and warrants further investigation due to the complex protein secretion and metabolic mechanisms in this fungus.

The commonly adopted tactic for protein expression is amplifying the copy number of target genes. A precise increase of four-fold enzyme activity (869.86 U/mL) was reported upon utilizing the CRISPR-HDR system to insert two or three copies of glucose oxidase (*goxC*) into the *amyA* and *glaA* sites [13]. Similarly, a multi-copy knock-in expression strategy using CRISPR/Cas9 was used to enhance TreM trehalase production (1943.06 U/mL) in *A. niger* [14]. To this end, we developed a double-copy expression cassette for monellin, which was integrated into the *aamA* locus and *amyA* locus. This integration resulted in 5MH, which expressed 4.1-fold more monellin than MH. We subsequently deleted both *pepA* and *pepB* in 5MH, generating DLpA-pB. After optimizing the fermentation medium, we achieved 0.284 mg/L of monellin in the shake flask supernatant.

Taken together, even though we explored different strategies to enhance the expression of the heterologous small protein monellin in *A. niger*, the final results were less than satisfactory. When the heterologous protein is secreted into the extracellular medium, eliminating its degradation by extracellular proteases and compounds in the culture medium can effectively solve this problem. However, heterologous proteins are heavily degraded during the process from the nucleus to the extracellular compartment, as reported in the literature [2,4,5,46], we are still unclear about the specific folding and processing mechanism for heterologous proteins in the cell, and there are few studies in this area. As a bottleneck problem for the expression system of *A. niger* today, improving the expression of heterologous proteins still needs further in-depth study. In future work, we think we can try to improve the expression of monellin in three aspects. The first aspect is improving the stability of mRNA. In this study, to improve the stability of mRNA, we performed codon optimization of monellin using an inducible strong promoter and its signal peptide in *A. niger*. However, as described previously, the knockdown of key ERAD pathway genes did not improve monellin expression. It has been reported that intron deletion almost completely inhibited the expression of endogenous acid lipase in *A. niger* [47]. The introduction of two introns from *A. niger* fructose-1,6-bisphosphatase resulted in the production of 1.8 mg/L of human erythropoietin by *A. niger* without detectable target protein prior to introduction [48]. As reported, with more research on introns, “intron engineering” may be an effective method to increase the expression of these non-fungal origin proteins [4]. In addition, the protein secretion mechanism of *A. niger* was investigated in depth in combination with RNA-seq technology. In filamentous fungi, proteins completing endoplasmic reticulum processing form the envelope protein complex COPII with vesicles, which are transported cis from the endoplasmic reticulum to the Golgi apparatus [49], then transported to the plasma membrane via secretory vesicles or granules, and finally released outside the eukaryotic cell, and this secretion process is a key factor affecting the amount of heterologous protein expression. There is less research on *A. niger* for this aspect, but it can be a good research direction with the protein transport pathway engineering of yeast as a reference [50]. The final aspect is morphological engineering. A number of studies have been reported on the morphology of *A. niger* [51,52], which can be triggered to undergo hyperbranching of mycelium and form a large number of mycelial tips, which facilitates protein secretion [8]. Addressing the final step of protein secretory expression in Aspergillus niger will have a direct impact on the amount of heterologous protein production.

## 5. Conclusions

This study successfully expressed recombinant monellin in *A. niger* for the first time. The low-expressed monellin was then fused with the HiBiT-Tag using bioluminescence to construct a model for studying the ultra-low-level expression of heterologous proteins in *A. niger*. Various strategies were explored for optimizing the expression of monellin, including increasing its copy number, fusing it with the endogenous highly expressed glycosylase *glaA*, mitigating extracellular protease degradation, overexpressing molecular chaperones, attenuating the ERAD pathway, and enhancing phospholipid synthesis in cellular biofilms. The goal of this study focused on enhancing the expression of the heterologous protein monellin and was intended to serve as a reference for the expression of other heterologous proteins in *A. niger*.

## Figures and Tables

**Table 1:** The *A. niger* strains used in this study.

*A. niger* Strain	Parental Strain	Description	Reference
WT	SH-2	*∆kusA, ∆pyrG*	[30]
MH	WT	one copy of monellin, *∆glaA*	This study
DL*pepA*	MH	*∆pepA*	This study
DL*pepB*	MH	*∆pepB*	This study
DL*derA*	MH	*∆derA*	This study
DL*hrdC*	MH	*∆hrdC*	This study
OE*pah1*	MH	*oepah1*	This study
OE*ino2*	MH	*oeino2*	This study
OE*opi3*	MH	*oeopi3*	This study
OE*pdiA*	MH	*oepdiA*	This study
OE*bipA*	MH	*oebipA*	This study
OE*sed5*	MH	*oesed5*	This study
3MH	MH	Three copies of monellin, *∆amyA*	This study
2M-glaA	MH	Fusion of glaA and monellin, *∆amyA*	This study
5MH	3MH	Five copies of monellin, *∆aamA*	This study
DL*pepA-2*	5MH	*∆pepA*	This study
DL*pepB-2*	5MH	*∆pepB*	This study
DLpA-pB	5MH	*∆pepA, ∆pepB*	This study
DLpAOEin	5MH	*∆pepA,* oe *ino2*	This study
DLpBOEop	5MH	*∆pepB,* oe *opi3*	This study
DLpA-pBOEin	5MH	*∆pepA, ∆pepB,* oe *ino2*	This study
DLpA-pBOEop	5MH	*∆pepA, ∆pepB,* oe *opi3*	This study
DLpA-pBOEin-op	5MH	*∆pepA, ∆pepB,* oe *ino2,* oe *opi3*	This study

Note: DL: delete, oe: overexpression, ∆: Knockout.

**Table 2:** The expression cassette plasmids and CRISPR/Cas9 plasmids constructed in this study.

*A. niger* Strain	Gene Expression Cassette	CRISPR/Cas9 Plasmids	Reference
		pFC330,	[31]
		pFC332-Dual loci	[13]
MH	pMD18T-P*glaA*-M-T*glaA*	pFC330-1(PAfU6-*glaA*sgRNA-TU6)	This study
DL*pepA*	pMD18T-U*pepA*-D*pepA*	pFC330-2(PAfU6-*pepA*sgRNA-TU6)	This study
DL*pepB*	pMD18T-U*pepB*-D*pepB*	pFC330-3(PAfU6-*pepB*sgRNA-TU6)	This study
DL*derA*	pMD18T-U*derA*-D*derA*	pFC330-4(PAfU6-*derA*sgRNA-TU6)	This study
DL*hrdC*	pMD18T-U*hrdC*-D*hrdC*	pFC330-5(PAfU6-*hrdC*sgRNA-TU6)	This study
OE*pah1*	pMD18T-P*aamA-pha1-*T*aamA*	pFC330-6(PAfU6-*aamA*sgRNA-TU6)	This study
OE*ino2*	pMD18T-P*aamA-ino2*-T*aamA*	pFC330-6(PAfU6-*aamA*sgRNA-TU6)	This study
OE*opi3*	pMD18T-P*aamA-opi3-*T*aamA*	pFC330-6(PAfU6-*aamA*sgRNA-TU6)	This study
OE*pdiA*	pMD18T-P*aamA-pdiA-*T*aamA*	pFC330-6(PAfU6-*aamA*sgRNA-TU6)	This study
OE*bipA*	pMD18T-P*aamA-bipA-*T*aamA*	pFC330-6(PAfU6-*aamA*sgRNA-TU6)	This study
OE*sed5*	pMD18T-P*aamA-sed5-*T*aamA*	pFC330-6(PAfU6-*aamA*sgRNA-TU6)	This study
3MH	pMD18T-Pna/TPI-M-T*glaA*-T*tef*-M-Pna/TPI-D*amyA*	pFC330-7(PAfU6-amyAsgRNA-TU6)	This Study
2M-*glaA*	pMD18T-P*aamA*-*glaA*-M-T*aamA*	pFC330-6(PAfU6-*aamA*sgRNA-TU6)	This study
5MH	pMD18T-P*aamA*-M-T*glaA*-Ttef-M-Pna/TPI-T*aamA*	pFC330-6PAfU6-*aamA*sgRNA-TU6	This study
DL*pepA*-2	pMD18T-U*pepA*-D*pepA*	pFC330-2(PAfU6-*pepA*sgRNA-TU6)	This study
DL*pepB*-2	pMD18T-U*pepB*-D*pepB*	pFC330-3(PAfU6-*pepB*sgRNA-TU6)	This study
DLpA-pB	pMD18T-U*pepA*-D*pepA* pMD18T-U*pepB*-D*pepB*	pFC330-8(PAoU6-*pepB*sgRNA-TU6-PAfU6-*pepA*sgRNA-TU6)	This study
DLpAOEin	pMD18T-U*pepA*-P*aamA*-*ino2*-T*pepA*	pFC330-2(PAfU6-*pepA*sgRNA-TU6)	This study
DLpBOEop	pMD18T-U*pepB*-P*aamA*-*opi3*-T*pepB*	pFC330-3(PAoU6-*pepB*sgRNA-TU6)	This study
DLpA-pBOEin	pMD18T-U*pepA*-P*aamA*-*ino2*-T*pepA* pMD18T-U*pepB*-D*pepB*	pFC330-8(PAoU6-*pepB*sgRNA-TU6-PAfU6-*pepA*sgRNA-TU6)	This study
DLpA-pBOEop	pMD18T-U*pepA*-D*pepA* pMD18T-U*pepB*-P*aamA*-*opi3*-T*pepB*	pFC330-8(PAoU6-*pepB*sgRNA-TU6-PAfU6-*pepA*sgRNA-TU6)	This study
DLpA-pBOEin-op	pMD18T-U*pepA*-P*aamA*-*ino2*-T*pepA* pMD18T-U*pepB*-P*aamA*-*opi3*-T*pepB*	pFC330-8(PAoU6-*pepB*sgRNA-TU6-PAfU6-*pepA*sgRNA-TU6)	This study

Note: DL: delete, oe: overexpression, ∆: Knockout.

**Table 3:** Expression of monellin in different Aspergillus niger strains compared to MH.

*A. niger* Strain	Parental Strain	Description	Compared with MH
MH	WT	one copy of monellin, *∆glaA*	1
DL*pepA*	MH	*∆pepA*	3.7 times increase
DL*pepB*	MH	*∆pepB*	2.35 times increase
DL*derA*	MH	*∆derA*	No impact
DL*hrdC*	MH	*∆hrdC*	Decreased by 22.3%
OE*pah1*	MH	oe *pah1*	No impact
OE*ino2*	MH	oe *ino2*	3.67 times increase
OE*opi3*	MH	oe *opi3*	1.90 times increase
OE*pdiA*	MH	oe *pdiA*	No impact
OE*bipA*	MH	oe *bipA*	No impact
OE*sed5*	MH	oe *sed5*	Increased by 22.3%
3MH	MH	Three copies of monellin, *∆amyA*	2.70 times increase
2M-glaA	MH	Fusion of glaA and monellin, *∆amyA*	2.80 times increase
5MH	3MH	Five copies of monellin, *∆aamA*	4.10 times increase
DL*pepA-2*	5MH	*∆pepA*	43.87 times increase
DL*pepB-2*	5MH	*∆pepB*	21.65 times increase
DLpA-pB	5MH	*∆pepA, ∆pepB*	55.51 times increase
DLpAOEin	5MH	*∆pepA,* oe *ino2*	9.43 times increase
DLpBOEop	5MH	*∆pepB,* oe *opi3*	25.01 times increase
DLpA-pBOEin	5MH	*∆pepA, ∆pepB,* oe *ino2*	14.27 times increase
DLpA-pBOEop	5MH	*∆pepA, ∆pepB,* oe *opi3*	28.17 times increase
DLpA-pBOEin-op	5MH	*∆pepA, ∆pepB,* oe *ino2,* oe *opi3*	23.78 times increase

Note: DL: delete, oe: overexpression, ∆: Knockout.

## Data Availability

All relevant data generated or analyzed during this study are included in this article.

## Supplementary Materials

The following supporting information can be downloaded at: https://www.mdpi.com/article/10.3390/jof9050528/s1, Figure S1. Schematic diagram of the CRISPR/Cas9 plasmid used in this study; Figure S2. Upstream and downstream positioning of the MH genome; Figure S3. Schematic diagram of gene deletion and overexpression; Figure S4. The relative transcription of ino2 and opi3 in each strain; Table S1. The medium and its components used in this study; Table S2. The amino acid sequence and optimized base sequence of monellin expressed in this study; Table S3. Primers for CRISPR/Cas9 plasmid construction in this study; Table S4. The gene-specific primers used for qRT-PCR analysis; Table S5. Reaction mixture for qRT-PCR analysis; Table S6. The condition of qRT-PCR cycle; Table S7. Primers used in the identification of transformants in this study.
