# Supplementary material for: Exploration of the Strategy for Improving the Expression of Heterologous Sweet Protein Monellin in Aspergillus niger
Source: J Fungi (Basel). 2023 Apr 29;9(5):528. doi: 10.3390/jof9050528 (PMC10218968; doi:10.3390/jof9050528)
Supplement: Supplementary file 1 [file jof-09-00528-s001.zip › jof-2365626-supplementary.pdf]

Supplementary imformation for

Exploration of the Strategy for Improving the Expression of Heterologous Sweet Protein Monellin in *Aspergillus niger*

Ke Li, Jun-Wei Zheng, Le-Yi Yu, Bin Wang and Li Pan *

School of Biology and Biological Engineering, South China University of Technology, Guangzhou Higher Education Mega Center, Guangzhou 510006, China; 202020148715@mail.scut.edu.cn (K.L.);
20710106321@mail.scut.edu.cn (J.-W.Z.);
201911008179@mail.scut.edu.cn (L.-Y.Y.);
btbinwang@scut.edu.cn (B.W.)
* Correspondence: btlipan@scut.edu.cn

Catalogue of supplementary materials

Table S1 The medium and its components used in this study

Table S2. The amino acid sequence and optimized base sequence of monellin expressed in this study

Table S3. Primers for CRISPR/Cas9 plasmid construction in this study

Table S4. The gene-specific primers used for qRT-PCR analysis

Table S5. Reaction mixture for qRT-PCR analysis

Table S6. The condition of qRT-PCR cycle

Table S7. Primers used in the identification of transformants in this study

Figure S1. Schematic diagram of the CRISPR/Cas9 plasmid used in this study

Figure S2. Upstream and downstream positioning of the MH genome

Figure S3. Schematic diagram of gene deletion and overexpression

Figure S4. The relative transcription of *ino2* and *opi3* in each strain.

Table S1 The medium and its components used in this study

media	Components and their contents
Luria-Bertani (LB)	peptone 1% (w/v), yeast extract 0.5% (w/v), and NaCl 1% (w/v)
Czapek-Dox medium (CD)	glucose 2% (w/v), $NaNO_3$ 0.3% (w/v), KCl 0.2% (w/v), $MgSO_4$-$7H_2O$ 0.05% (w/v), KH_2PO_4 0.1% (w/v), $FeSO_4$-$7H_2O$ 0.001% (w/v), and Agar 0.05% (w/v)
The hyperosmotic CD medium	sucrose 35% (w/v), $NaNO_3$ 0.3% (w/v), KCl 0.2% (w/v), $MgSO_4$-$7H_2O$ 0.05% (w/v), KH_2PO_4 0.1% (w/v), and $FeSO_4$-$7H_2O$ 0.001% (w/v)
DPY medium	glucose 2% (w/v), peptone 1% (w/v), yeast extract 0.5% (w/v), KH_2PO_4 0.5% (w/v), and $MgSO_4$-$7H_2O$ 0.05% (w/v)
Starch fermentation medium	starch 5% (w/v), corn pulp 3% (w/v), and soybean meal powder 2% (w/v)

Table S2. The amino acid sequence and optimized base sequence of monellin expressed in this study

The amino acid sequence and optimized base sequence of monellin
Green: Signal peptide of *glaA*<br>Blue: 8*His tag<br>Red: amino acid sequence of monellin<br>Black: linker<br>Orange: the HiBiT-tag<br>MSFRSLLALSGLVCTGLAHHHHHHHHGEWEIIDIGPFTQNLGKFAVDEENKI<br>GQYGRLTFNKVIRPCMKKTIYEENGFREIKGYEYQLYVYASDKLFRADISED<br>YKTRGRKLLRFNGPVPPPGSGSGVSGWRSFKKIS*<br>ATGTCGTTCCGATCTCTACTCGCCCTGAGCGGCCTCGTCTGCACAGGGTT<br>GGCACATCACCACCACCATCATCATCACGGGGAATGGGAGATCATCGACA<br>TTGGCCCCTTCACGCAGAACTTGGGAAAATTTGCAGTGGATGAGGAGAA<br>CAAAATAGGACAATACGGCAGACTTACATTCAACAAGGTCATTCGCCCTT<br>GCATGAAGAAGACCATCTACGAAGAGAATGGCTTCCGAGAAATCAAGGG<br>TTATGAGTACCAGCTATATGTCTATGCCTCGGATAAGCTCTTCCGCGCTGA<br>TATTTCCGAAGACTACAAGACTCGTGGGCGGAAACTGCTGAGGTTTAATG<br>GTCCGGTTCCCCCACCTGGCAGTGGATCTGGAGTGAGCGGCTGGCGGTC<br>GTTCAAGAAGATTAGCTAA

Table S3. Primers for CRISPR/Cas9 plasmid construction in this study

Primer	Sequences (5'→3')	Description
PAfU6-F	gtttccgctgagggtttaatttaattaagcaggcg gttgcaagcgatc	Amplification of PAfU6
PAfU6-R	ctttcattcttacagacctct	Amplification of PAfU6
PAoU6-F	tctgcggaacatatactgggcccgggaatggttc acttctctttagaaa	Amplification of PAoU6
TU6-R	ctgtctcggctgaggtcttaagcagctctatatca cgtgacg	Amplification of TU6
*amyA*sgRNA-F	ggtctgtaagaatgaaagtcttcggcccttcatga gaggttttagagctagaaatagc	Amplification of *amyA*sgRNA
*glaA*sgRNA-F	ggtctgtaagaatgaaaggttgagcaacgaagc gaccggttttagagctagaaatagc	Amplification of *glaA*sgRNA
*aamA*sgRNA-F	ggtctgtaagaatgaaaggcgatggtgaagctta ccagttttagagctagaaatagc	Amplification of *aamA*sgRNA
*pepA*sgRNA-F	ggtctgtaagaatgaaagggtgccccagagcgt gaagggttttagagctagaaatagc	Amplification of *pepA*sgRNA
*pepB*sgRNA-F	ggtctgtaagaatgaaaggatcggcgacggcta caccagttttagagctagaaatagc	Amplification of *pepB*sgRNA
*derA*sgRNA-F	ggtctgtaagaatgaaagtgaatgccatcagaac ccaggttttagagctagaaatagc	Amplification of *derA*sgRNA
*hrdC*sgRNA-F	ggtctgtaagaatgaaaggcttgggtttatgtatg caagttttagagctagaaatagc	Amplification of *hrdC*sgRNA

Note*: The red marked part of the table are the 20bp protospacers

Table S4. The gene-specific primers used for qRT-PCR analysis

Primer sequence（5'-3'）		Description
gpdA-F	TCTGCTCCTTCCGCTGATG	Reference gene
gpdA-R	CCCTCAACGATGCCGAAC	Reference gene
Opi3-F	GGCTGCTACTTCCTCGCTG	Target gene *opi3*
Opi3-R	AGGGGCATAATAAGGTTGGTC	Target gene *opi3*
Ino2-F	AGAAGACGACGACCATACGG	Target gene *ino2*
Ino2-R	GGGCTTTGGACCTTGAGAA	Target gene *ino2*
bipA-F	ACCGTCCCCGCCTACTTC	Target gene *bipA*
bipA-R	CACCAGTCTTGTCCAATCCGT	Target gene *bipA*
pdiA-F	CCAAGATGTTCGGTGCCC	Target gene *pdiA*
pdiA-R	CCTGGTCATAGGGGTATTTGG	Target gene *pdiA*
Pha1-F	CAGTCACCGACGCCAGAA	Target gene *pha1*
Pha1-R	TGTTCGCACCAGGCTTCA	Target gene *pha1*
Sed5-F	CGCTCCGATTCCCCACTT	Target gene *sed5*
Sed5-R	ATTGCCTCACCACGCCCT	Target gene *sed5*

Table S5. Reaction mixture for qRT-PCR analysis

Reaction Component	Concentration	Volume(μl)
SybrGreen qPCR Master Mix	2X	10
Primer F (10 μM)	10 μM	0.4
Primer F (10 μM)	10 μM	0.4
ddH20	/	7.2
Template (cDNA)	/	2
Total	/	20

Table S6. The condition of qRT-PCR cycle

Thermal Cycler	Times and Temperatures			Dissociation
	Initial Steps	Each of 45 cycles		
		Melt	Anneal/Extend	Accoding to instrument guidelines
QuantStudio™ 1 Plus Fluorescence quantitative PCR instrument	HOLD	CYCLE		
	3 min 95°C	15 s 95°C	30 s 60°C	

Table S7. Primers used in the identification of transformants in this study

Primer	Sequences (5'→3')	Function description
Monecds-F	TGGCCCCTTCACGCAGAAC	Monellin DNA sequence validation
Monecds-R	AAACCTCAGCAGTTTCCGCC	Monellin DNA sequence validation
*glaA*UY-F	GGACCTTATTCGCCTTGTTT	Location of monellin in the *glaA* locus
*glaA*UY-R	GCGAATGACCTTGTTGAATG	Location of monellin in the *glaA* locus
*glaA*DY-F	CGGCAGACTTACATTCAACA	Location of monellin in the *glaA* locus
*glaA*DY-R	TTTTCGTTGAAGCCTTGAGC	Location of monellin in the *glaA* locus
*pepA*cds-F	GTCACCACCAACAAGCAGG	Identify whether *pepA* has been deleted
*pepA*cds-R	CGGCGAAAAGGGGAGAGTC	Identify whether *pepA* has been deleted
*pepB*cds-F	ACTGGCGAGTTCACTGTCC	Identify whether *pepB* has been deleted
*pepB*cds-R	TACCAGGCATCGTAGGAAG	Identify whether *pepB* has been deleted
*derA*cds-F	CTCGCCATTTCTCTCCCTT	Identify whether *derA* has been deleted
*derA*cds-R	CGTGAAGCGAAGGGTAAAC	Identify whether *derA* has been deleted
*hrdC*cds-F	ATGCTTGGGTTTATGTATG	Identify whether *hrdC* has been deleted
*hrdC*cds-R	CCTTCACCATAAACACCAC	Identify whether *hrdC* has been deleted
*aamA*cds-F	TCCACCCATACTGCCTGAT	Verify whether *aamA* has been replaced
*aamA*cds-F	GTAGAGGTTGCTGATGCTG	Verify whether *aamA* has been replaced
*aamA*UY-F	CAACGGTTTGCAGGGTCAT	Location of gene overexpression cassette
*aamA*DY-R	AGGGACATGCTGAGAACCA	Location of gene overexpression cassette
*amyA*UY-F	CTAGCTGCACTAGGAAGCAAT	Location of 3M in the *amyA* locus
*amyA*UY-R	ACTCCGTCACCAAGATCACTAT	Location of 3M in the *amyA* locus
*amyA*DY-F	CAGCGGATTTGAAGTTCAGG	Location of 3M in the *amyA*

		locus
*amyA*DY-R	AGCGCTTTTGCTTCCTTGCA	Location of 3M in the *amyA* locus
*pepA*UY-F	CCAGTCTCACGGCATCAGT	Location of *ino2* in the *pepA* locus
*pepA*UY-R	AGGCTGCGGACTATTCTGG	Location of *ino2* in the *pepA* locus
*pepA*DY-F	CAAAGCCCGCACGAGAGAA	Location of *ino2* in the *pepA* locus
*pepA*DY-R	CAGTCCCCGAGACGGTTTA	Location of *ino2* in the *pepA* locus
*pepB*UY-F	GCAACATTCGTCAAGGAGT	Location of *opi3* in the *pepB* locus
*pepB*UY-R	AAGGGAGCGTAGAAGAAGA	Location of *opi3* in the *pepB* locus
*pepB*DY-F	ATGGTAGAAACCCACTGAC	Location of *opi3* in the *pepB* locus
*pepB*DY-R	TCCATTCTTTTGCTTACAT	Location of *opi3* in the *pepB* locus

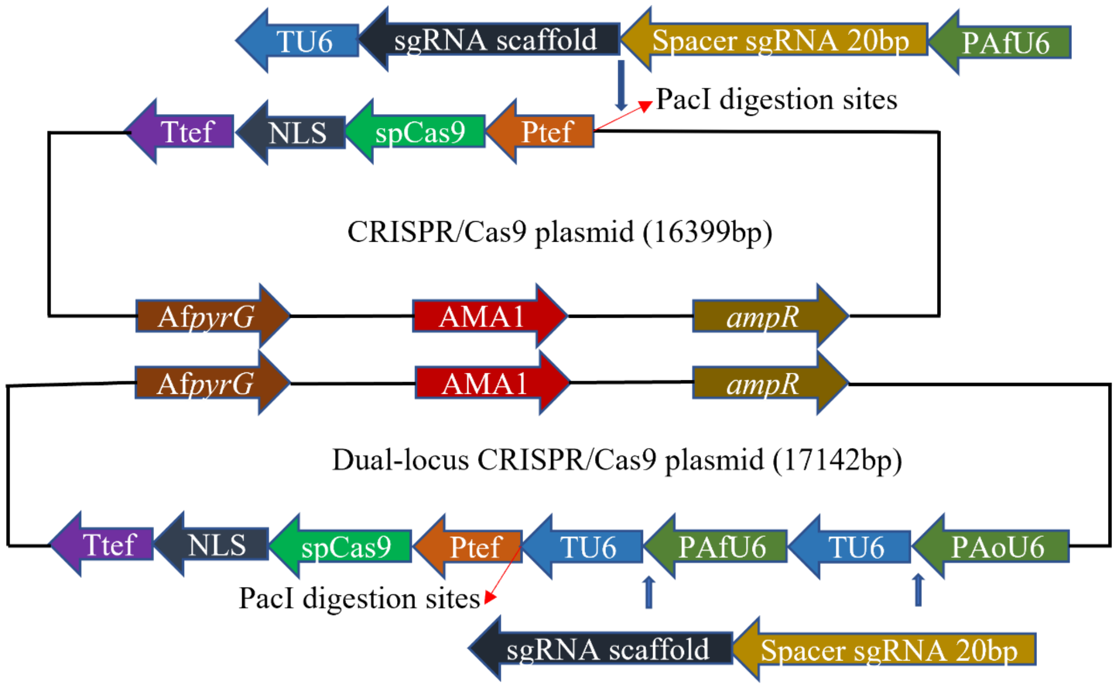

Figure S1. Schematic diagram of the CRISPR/Cas9 plasmid used in this study

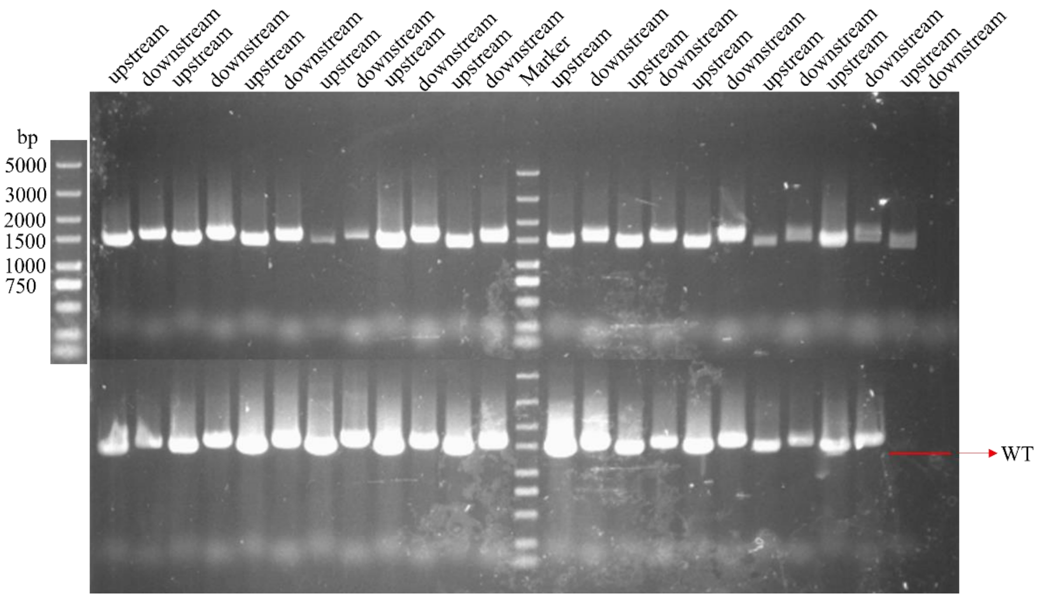

Figure S2. Upstream and downstream positioning of the MH genome

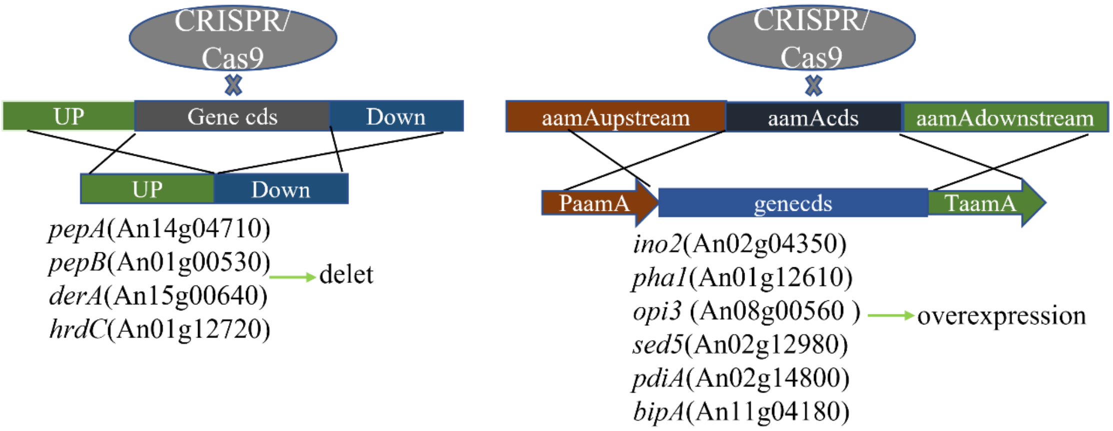

Figure S3. Schematic diagram of gene deletion and overexpression

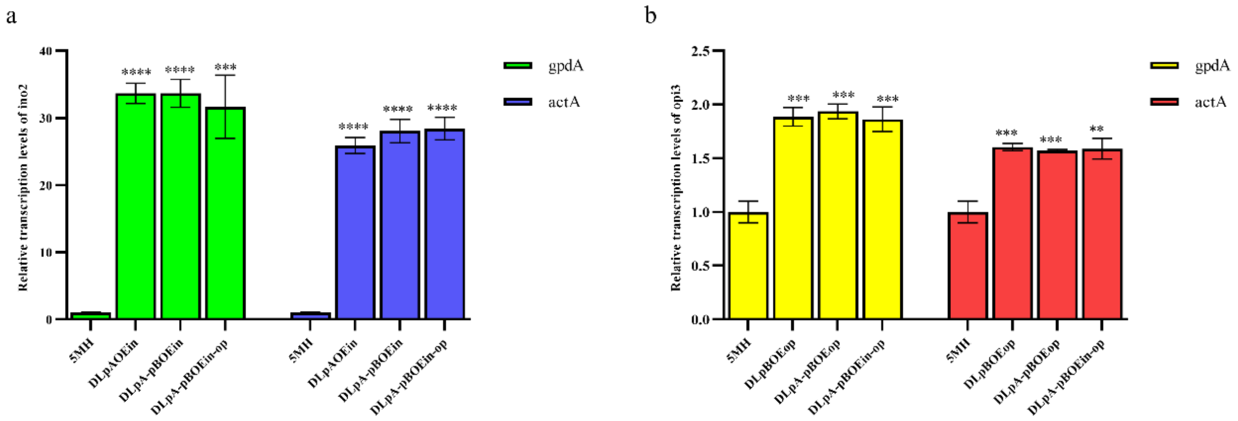

Figure S4. The relative transcription of *ino2* and *opi3* in each strain.
